# Condom-Protected Sex and Minority Stress: Associations with Condom Negotiation Self-Efficacy, “Passing” Concerns, and Experiences with Misgendering among Transgender Men and Women in Brazil

**DOI:** 10.3390/ijerph18094850

**Published:** 2021-05-01

**Authors:** Ramiro Figueiredo Catelan, Alexandre Saadeh, Maria Inês Rodrigues Lobato, Daniel Augusto Mori Gagliotti, Angelo Brandelli Costa

**Affiliations:** 1 Psychology Graduate Program, Pontifical Catholic University of Rio Grande do Sul, Porto Alegre 90619-900, Brazil; angelo.costa@pucrs.br; 2 Institute of Psychiatry, Federal University of Rio de Janeiro, Rio de Janeiro 22290-140, Brazil; 3 Department of Psychology, Pontifical Catholic University of São Paulo, São Paulo 05014-901, Brazil; alesaadeh@yahoo.com.br; 4 Faculty of Medicine Clinics Hospital, University of São Paulo, São Paulo 05403-000, Brazil; danimori_medusp@yahoo.com.br; 5 Hospital de Clínicas de Porto Alegre, Porto Alegre 90035-903, Brazil; mirlobato@gmail.com

**Keywords:** sexual behaviour, transgender, condom use, condom negotiation, risky sexual behaviour, prejudice, HIV, minority stress

## Abstract

This cross-sectional exploratory study aims to verify associations between condom-protected sex, condom negotiation self-efficacy, self-esteem, and four minority stressors (experiences with misgendering, “passing” concerns, anticipated prejudice, and perceived prejudice) among transgender men (TM) and transgender women (TW). 260 individuals (192 TW and 68 TM) residing in two Brazilian states participated in the study. Data was collected online and in two hospital programs for transgender people and included sociodemographic data, condom-protected sex, the Trans-Specific Condom/Barrier Negotiation Self-Efficacy (T-Barrier) Scale, the Rosenberg Self-Esteem Scale, and four minority stressors. Measures that were significantly associated with condom-protected sex were tested as independent variables in a linear regression model. The main results suggest that lower condom negotiation self-efficacy, higher “passing” concerns, and higher experiences with misgendering were predictors of lower frequency of condom-protected sex. These negative outcomes were found among both TM and TW, which justifies their inclusion in public health policies. Structural strategies and clinical interventions are suggested to address condom negotiation self-efficacy and “passing” concerns in transgender populations.

## 1. Introduction

Condom-protected sex (CPS) refers to individual’s consistent use of condoms during sexual intercourse, while condom negotiation self-efficacy (CNSE) refers to the confidence to assertively ensure condom use during sexual intercourse, preventing unwanted non-use. The literature shows that CPS and CNSE are associated with consistency/regularity of use and act as protectors against HIV and other sexually transmitted infections (STIs) in the cisgender population, that is, people whose gender identity is aligned with the sex designated at birth [1,2,3,4,5].

Sexual coercion, abusive relationships and exposure to intimate partner violence are some of the factors associated with unprotected sex without condoms and decreased CNSE capacity with sexual partners. These experiences have been widely investigated in cisgender people, particularly cisgender women [6,7,8,9]. Studies also point out that previous negative experiences with condom use are associated with irregular and decreased use [10,11]. One of the factors related to a decrease in condom-use intention in this population is low levels of self-esteem, that is, the presence of negative emotional responses about themselves [12,13,14].

There are similar studies with samples of transgender people, that is, those whose gender identity is not aligned with the sex designated at birth, a population commonly exposed to numerous inequities and vulnerabilities. In research conducted in the Dominican Republic with 78 transgender women (TW) who were sex workers, exposure to violence increased the chances of not using condoms by up to 4.75×, highlighting the vulnerability to which this population is exposed [15]. The weight of HIV is high among this population and associated with exposure to prejudice and other structural factors, which reinforces the importance of creating public policies that address the HIV care cascade, including the use of condoms [16].

Also, low self-esteem, history of forced sex, and use of crack and cocaine can contribute to inconsistent condom use among transgender individuals [17]. Syndemic vulnerabilities, such as alcohol abuse, depressive symptoms, and childhood sexual abuse, can negatively affect the ability to get condoms, use them correctly, and negotiate their use during sex [18]. Self-efficacy in condom use consistency can act as a protective factor against unprotected anal sex in commercial sexual relations/prostitution [19]. Negotiating condom use can be difficult for TW, who are less likely to use condoms with their partners, as protected sexual behaviors can lead to both the expectation of relational rejection/disruption and physical or sexual violence, which tends to cause intense psychological distress [20].

The minority stress model, originally developed by Meyer [21], is one of the explanations with more empirical support for the inequalities present in the lives of transgender individuals [22]. Transgender minority status leads to increased exposure to distal stressors, external to the individual, such as direct experiences of violence, family rejection, restriction of civil rights, discrimination in health services, and experiences with misgendering (when people do not recognize the true/actual gender of transgender people and may treat them with wrong pronouns intentionally or unintentionally and/or identify them as transgender due to their gender expression) [23]. Systematic exposure to distal stressors leads to experiencing proximal stressors, which are the individual’s internal processes and perceptions. This includes perceived prejudice (perception of discrimination directed at them for being transgender), anticipated prejudice (expectation of rejection/discrimination for being transgender), “passing” concerns (desire to not appear to be a transgender person and/or seeking to have a gender expression similar to that of a cisgender person), among others [24,25,26]. The combination of the two sets of stressors leads to negative psychological outcomes, which are already well documented in the literature [27,28,29].

In Brazil, where the research described in this study was conducted, the transgender population is systematically exposed to vulnerability and social marginalization, which leaves them susceptible to harmful situations. A recent study found that around 67.20% of transexual individuals have depressive symptoms, a high rate considering that the general prevalence among the Brazilian population is 5.8%. The prevalence of suicidal ideation was 67.72%, while that of suicide attempts was 43.12%. Among those who attempted suicide, 80.50% reported associating their attempt with being a trans person. The aversive context to which trans individuals are exposed is illustrated by the fact that 67.50% of the sample reported having avoided a location for fear of being attacked or thrown out for being transgender [30].

The murder rates in this population in Brazil are considered to be the highest in the world, with an average of 118.2 murders/year between 2008 and 2019 [31]. Between 33.85% and 45.16% of these individuals are not currently employed, most of them reporting having jobs denied due to their gender identity [32]. A study on the health conditions of TW in the state of Rio de Janeiro found that the participants had low income (62.0% lived on less than USD10.00/day), demonstrated a very high involvement in sex work (78.6%) and reported a higher occurrence of sexual (46.3%) and physical (54.0%) violence [33]. There is scarce data on transgender men (TM), a neglected population in research settings whose environmental conditions and associated vulnerabilities are not well documented in Brazilian context.

Minority stressors such as expectations of rejection and concealment of gender identity are associated with risky behaviors for HIV infection, such as having sex without a condom [34]. Little is known about the association between CPS and variables such as CNSE, self-esteem and some specific minority stressors, such as experiences with misgendering, “passing” concerns, anticipated prejudice, and perceived prejudice, which can act as sexual vulnerability factors in transgender individuals.

The relationship between prejudice (whether anticipated or the suffering of discrimination) acts as stressors that can reduce mental health, high self-esteem and therefore self-care. In addition, they can negatively impact the identity and self concept through the internalization of prejudice. The experiences of misgendering and “passing” concerns in addition to being stressors in their own right (worsening mental health) are associated with the previous process of more general minority stress, increasing its effects. As we already know that people with worse mental health use less condoms, and since minority stress (including their gender aspects) affects mental health, we intend to investigate this relationship.

The main objective of this study is to verify associations between CPS, CNSE, self-esteem and four dimensions of minority stress (experiences with misgendering, “passing” concerns, anticipated prejudice, and perceived prejudice) in transgender individuals. The specific objectives are (1) to investigate differences between TM and TW in measures that assess CPS, CNSE, self-esteem and minority stress dimensions; (2) to verify the links between the surveyed variables; and (3) to analyze possible predictors of CPS. Our central hypothesis is that CNSE and self-esteem are positively associated with CPS, while the four minority stress dimensions are inversely related to CPS.

## 2. Materials and Methods

### 2.1. Procedure

The project “Saúde da População Trans” (“Health of the Trans Population”), which took place between 2014 and 2015, was a cross-sectional exploratory project conducted through online and face-to-face surveys, built with contributions from the medical and transgender communities, to assess various aspects of the health of transgender people living in two Brazilian states. The research was modeled after the TransPULSE project, which was one of the first large-scale studies that addressed the healthcare needs and vulnerabilities of transgender people. The original project sought to improve the quality of life of transgender people in Ontario, Canada, by measuring levels of social exclusion and its impact on physical and mental health. Studies derived from the “Trans health” project include the impact of minority stress on suicide ideation, suicide attempts and depressive symptoms [30]; prevalence of labor market discrimination [32]; an assessment of transgender-specific healthcare needs in the Brazilian context [35]; HIV-related healthcare needs [36]; the impact of family support on the process of gender affirmation [37]; and the association between parental participation in transgender children’s life and discrimination experiences [38]. 

The project’s adaptation to the Brazilian context was an initiative of the Federal University of Rio Grande do Sul (UFRGS) in partnership with the University Hospital of the School of Medicine of the University of São Paulo (HCFMUSP). All subjects gave their informed consent for inclusion before they participated in the study. The study was conducted in accordance with the Declaration of Helsinki, and the protocol was approved by the Ethics Committee of UFRGS on June 6th, 2013 (protocol n. 14221513.4.0000.5334).

### 2.2. Participants

Since the Brazilian Unified Health System provides georeferenced care, patients seeking gender affirmation must access those procedures in the states in which they live. Data were collected in Rio Grande do Sul and São Paulo, two states that are pioneers in providing specialized services to transgender people since the implementation of the first Brazilian healthcare policies for this population. Both states have gender identity programs that provide body modification procedures at university hospitals. Patients of the two programs were voluntarily invited to complete an electronic version of the survey using tablets in the presence of the researchers. In this case, data collection took place at the hospital itself before or after the appointments. The questionnaire was also available on the Internet through an online Facebook adds that focused on trans people during two time periods: July–October 2014 and January–March 2015. The participants have not received neither payment incentive nor any direct benefit for joining the research.

For this study, the sample consisted of 260 people with negative HIV status (who were controlled from the original total sample), due to the differences that may exist in terms of sexual behavior between HIV-positive and HIV-negative people. Gender identity was assessed using the two-question method, and persons were considered eligible for participation if they reported a gender different from that assigned to them at birth. Based on their self-reported gender identity, participants were re-categorized as transgender women (TW), transgender men (TM), or gender-diverse people. TW were those who were designated as male at birth but identified as women, TW or *travestis*. *Travesti* is a culturally specific gender identity term in the Brazilian context. *Travestis* are transgender people who were assigned male at birth, but affirm a female performance and bodily form, although they typically do not undergo neovaginoplasty. Their gender identity varies; most identify as male, some idenfity as women, and others simply identify as *travestis*. TM were those who were assigned female at birth but identified as men or TM. 

Finally, gender-diverse persons were those who identified with a gender identity outside the binaries (male-female), such as queer, non-binary, a-gender, etc. Of the total number of participants, 73.8% identified as TW and 26.2% as TM. Due to an insufficient sample size, non-binary people were not included in this study. Most TW reported that they were sexually attracted to cisgender men (90.20%) and most TM reported that they were attracted to cisgender women (91.34%).

Race/skin color/ethnicity was determined using the Brazilian Institute of Geography and Statistics census categories: white, black, yellow (although considered offensive in some cultural contexts, it is the way that East Asians are categorized in the Brazilian census), and indigenous. One other category, *pardo*, was used, which commonly refers to Brazilians of a mixed race, typically a mixture of white, Afro- and native Brazilian. Approximately 70.8% of the participants self-declared as white, 62.3% completed high school, 70% self-declared to be single, and 69.6% were from São Paulo. The mean age was *M* = 28.70 years (*SD* = 8.66), in a range of 18 to 65 years. Besides 33,84% of TW and 45,16% of TM reported being unemployed at the time of data collection. Additional sociodemographic data can be found in Table 1.

### 2.3. Measures

#### 2.3.1. Sociodemographic

Sociodemographic characteristics such as gender identity, race, level of education, and geographic location were used in the descriptive analyses.

#### 2.3.2. Condom-Protected Sex (CPS)

Based on TransPULSE project, participants were asked if they or their partners wear a condom when having the following sexual practices: insertive oral sex, receptive oral sex, insertive penetration, and receptive penetration. Each sexual practice was measured on a 5-point Likert scale ranging from 1 (Always) to 5 (Never), which generated an overall score. Analyses included an overall score and each practice individually.

#### 2.3.3. Trans-Specific Condom/Barrier Negotiation Self-Efficacy (T-Barrier) Scale 

This scale was originally developed for TransPULSE project to assess condom negotiation self-efficacy (CNSE) among transgender populations [39]. We translated and adapted it for our study. Participants were asked the following question: “When I think about using condoms with a partner, I…”, whose answer options included seven items, rated on a 5-point Likert scale ranging from 1 (Not at all [worried]) to 5 (Very [worried]), which for scoring purposes had their items added together to form a total score: item 1 (“I can ask a new partner to use a condom”), item 2 (“I can ask a partner with whom I didn’t use a condom to start doing this”), item 3 (“I can refuse sex when I don’t have condoms available”), item 4 (“I can make a partner use a condom even when I’m drunk or drugged”), item 5 (“I can make a partner use a condom even when he/she doesn’t want to”), item 6 (“I can ask a partner who knows I am transgender to use condoms”), and item 7 (“I can ask any partner to use a condom”) (Cronbach’s α = 0.96).

#### 2.3.4. Rosenberg Self-Esteem Scale 

The Brazilian version of Rosenberg Self-Esteem Scale was used to assess self-esteem [40,41]. The scale consists of 10 items, classified on a 5-point Likert scale ranging from 1 (Never) to 5 (Always), whose items were added together to form an overall score for scoring purposes. Examples of items on this scale include “I can do things as well as most people”; “I have a positive attitude towards myself”; and “Overall, I am satisfied with myself” (Cronbach’s α = 0.88).

#### 2.3.5. Perceived Prejudice

The participants answered a question with several alternatives, based on the measures developed by Scheim and Bauer [42]. They were asked to point out any of the following situations of violence they had experienced: silent aggression; verbal aggression; physical intimidation and threats; physical aggression; sexual assault; sexual violence; and if they had ever been victims of some kind of violence.

#### 2.3.6. Anticipated Prejudice

The participants answered a question with several alternatives, based on the measures developed by Scheim and Bauer [42]. They were asked to point out any situations they had ever avoided for fear of aggression or of being expelled for being transgender. The options were: public transport; pharmacy; shopping malls or clothing stores; schools or colleges; traveling to other places; clubs or social groups; gyms; church, temples or other religious institutions; public restrooms; public spaces (for example parks, streets); restaurants or bars; and cultural centers.

#### 2.3.7. “Passing” Concerns

Participants were asked, based on the TransPULSE project, if they wanted other people to know they were trans. The possible answers were 1 (Yes), 2 (No), and 3 (I don’t care).

#### 2.3.8. Experiences with Misgendering

Participants were asked, based on the TransPULSE project, how often people who know them know they are transgender without being informed about it by them. The measurement was established on a 5-point Likert scale ranging from 1 (Always) to 5 (Never).

### 2.4. Data Analysis

Initially, the data were analyzed regarding their distribution patterns. Since there was only a slight asymmetry (asymmetry and kurtosis values close to zero), parametric statistical tests were carried out. In addition to descriptive calculations (measurements and standard deviations), t-tests were conducted to investigate possible differences between TM and TW regarding averages in CPS, CNSE, “passing” perception, self-esteem, anticipated prejudice, and perceived prejudice, as well as a chi-square test to verify the association between gender and “passing” concerns. False discovery rate (FDR) correction was applied to the different tests, considering the number of comparisons [43]. Subsequently, Pearson correlations were applied to the study’s continuous variables. The measures that were significantly associated with CPS in previous analyses were tested as independent variables in a linear regression model with the enter method. The model was considered adequate, as long as there was no violation of statistical assumptions (Durbin-Watson, normality of residuals, multicollinearity, among others).

## 3. Results

First, we will present data on the differences between TM and TW regarding the study variables (Table 2). TW had higher averages regarding condom use when they perform oral sex, when they receive penetration, and in CPS total score. Similarly, TW scored more on CNSE for different situations, as well as in self-esteem and experiences with misgendering. 

Differences between genders reached greater magnitude for the use of condoms when receiving penetration, CPS total score, and CNSE item 3 (“I can refuse sex when I don’t have condoms available”). As for “passing” concerns, this variable was not associated with gender (χ^2^ = 0.46; *p* > 0.05). Table 3 presents details on the Pearson correlation matrix.

Regarding “passing” concerns, it was found that the participants with these concerns obtained higher scores in some isolated items of CPS, and CPS total score. The largest effect magnitudes were found in the differences for CPS (receptive penetration) and CPS total score (Table 4).

Previous analyses have shown the differences between TM and TW. Due to an insufficient sample number to analyze TM and TW separately, a multiple linear regression model to group both genders was tested (Table 5). The variables associated with CPS total score (CNSE, self-esteem, experiences with misgendering, and “passing” concerns) were included as independent variables. Three predictors of CPS were found, with CNSE being the most strongly associated variable (β = 0.46), followed by “passing” concerns (β = 0.31), and experiences with misgendering (β = 0.19). This means that less self-efficacy in negotiating condom use, having more “passing” concerns, and experiences with misgendering can lead to decreased use of condoms.

## 4. Discussion

The first objective of the study was to investigate differences between TM and TW regarding the variables studied. “Passing” concerns not being associated with gender can demonstrate that this variable is present independently of gender, and it is a common concern for the transgender population in general [44]. The differences found for the CPS variable between TM and TW can be partially explained by the diversity of sexual practices. TW usually have penetrative sex (either insertive or receptive) in their sexual routine, while TM more often practice oral sex (receptive and insertive), masturbation and penetrative sex using sex toys, practices that, although they represent some risk of HIV/STIs infection (even if reduced), are usually done without using a condom [45]. In Brazil, TW are a target population for HIV/STIs public policies, which makes them get tested more often and be influenced to use condoms, especially sex workers, which may also explain the data found in the analysis of differences between genders [46]. Our findings suggest that Brazilian healthcare programs should expand their action scope to address practices specific to TM with education, prevention, and harm reduction campaigns.

The second objective was to assess the links between the study variables, taking CPS total score as the central parameter. No correlations were found between CPS total score and perceived prejudice, which contradicts one of our hypotheses. The minority stress model can help to understand this data. There is a difference between distal or external aspects (linked to experiences of violence, prejudice, or discrimination) and proximal or internal aspects (linked to the perception that violence, discrimination and prejudice were due to your identity or to the social group to which you belong) [21]. A transgender person may, for example, go through discriminatory experiences and think that this is not related to transphobic prejudice. Therefore, this experience, when not perceived as transphobic, probably does not contribute to the emotional dysregulation that can occur in people who perceive stigma, therefore, having no significant impact on sexual behavior, which could partially explain our results. This may be even more true in a context of endemic violence such as Brazil where the construction of identity should take into account a very high baseline level of violence arising from the environment.

We also need to take into account that we have two samples that may not contain the people who are in the most vulnerable situations. The sample of the hospital represents people who managed to navigate the system overcoming several barriers to get there and the sample of the internet represents people with economic conditions to have internet. Therefore, in more vulnerable segments of the transgender community, this association can be found. Brazil has public integral health policies for sexual and gender minorities. However, health services tend to focus excessively on gender affirmation procedures when providing treatment for transgender people, often neglecting other aspects of mental and sexual health that would be important to be addressed, as our study shows.

Contrary to what was expected, no correlations were found between CPS total score and anticipated prejudice. Despite some evidence demonstrating that the anticipation of prejudice and the expectation of rejection can contribute to risky behaviors [34], people may not use condoms due to other factors, such as low condom negotiation self-efficacy, more experiences with misgendering and, heightened “passing” concerns, as our data has shown. Variables that were not analyzed in this study, such as resilience, coping and connection with the community can also affect condom use. We also need to take into account that we have two samples that may not contain the people who are in the most vulnerable situations. The sample of the hospital represents people who managed to navigate the system overcoming several barriers to get there and the sample from the internet may represent people with good economic conditions. Therefore, in more vulnerable segments of the transgender community, this association could be found.

The third objective was to establish possible predictors of CPS. According to the final logistic regression model, CPS was significantly associated with CNSE, “passing” concerns, and experiences with misgendering, confirming the study hypotheses. The main association found was between CPS and CNSE, demonstrating that the lower the ability to negotiate condom use, the greater the chance of having sex without a condom. This data is compatible with those presented by research on condom negotiation self-efficacy and risky behaviors, which demonstrate that the lower the self-efficacy, the greater the probability of not using condoms in sexual intercourse [1,2,3,4,5]. Our study also suggests that even if there is high self-efficacy in ensuring the use of condoms in sexual intercourse, high levels of “passing” concerns and more experiences with misgendering can prevent people from negotiating condom use and having sex without a condom.

One of the main possible explanations for the association between CPS and CNSE is the expectation of romantic/sexual rejection and loneliness beliefs, which are commonly experienced by transgender individuals due to being transgender [47]. This could lead to low assertiveness in negotiating condom use in sexual intercourse, as these individuals commonly have loneliness beliefs and expect intimate rejection [48,49]. From this logic, if a hypothetic sexual partner of a transgender person insisted on having unprotected sex, for example, the fear of rejection and loneliness could be activated in that person, causing them to give in to pressure to maintain the relationship or avoid rejection, reducing their CNSE [34].

The data from our study has shown that having more “passing” concerns is related to being less likely to use condoms. This can be partially explained by the anxiety caused by this “passing” expectation, which can lead to not being able to ensure condom use in their relationships [50,51]. Bodily issues (such as negative self-image) that in some way may have to do with “passing” can cause the person to stop being careful in their sexual encounters in regards of condom use.

The literature has shown that “passing” concerns in transgender people are one aspect of rumination, a systematic pattern of thoughts directed at oneself as a maladaptive way of managing stressful situations, usually associated with negative moods. Rumination can have deleterious psychological effects on mental health, leading to a greater likelihood of developing mental disorders [30,52]. Evidence also suggests that negative psychological variables are associated with low condom use, which helps to explain our data [53].

It is important to note that, although many transgender people question the idea of “passing,” this happens from a social imposition, based on a normative binary assumption, causing great discomfort even to those who do not care about this logic, since there is immense social pressure to conform to standards [26]. It is a logic that is similar to sexual objectification, that is, internalization of social expectations about the sexualization of the female body, which can lead to greater self-monitoring of the body, shame and anxiety. Even cisgender women who refute and deny the objectification of their bodies suffer from external pressure that demands it from them [54]. This theoretical model of objectification, created based on the experiences of cisgender women, has been tested on TW, showing that objectification is associated with greater pressure to search for breast implants and more propensity to develop eating disorders [55]. Associated with transphobic prejudice, sexual objectification can increase body surveillance and body dissatisfaction [56]. In TM, sociocultural standards of attractiveness (one of the elements of sexual objectification) were directly associated with increased compulsion regarding physical exercise [57].

Contrary to our hypothesis, no association was found between CPS and self-esteem in the regression model, that is, it was expected that, regardless of the level of self-esteem, the low ability to negotiate condom use could decrease CPS frequency. A possible explanation for this is that, in the presence of other variables, self-esteem becomes less relevant, because the other variables studied are more representative or stronger. If a person has high self-esteem, for example, but goes through more misgendering experiences or has more “passing” concerns, there will still be a negative impact on condom use.

From the analyses, we found that having had more experiences with misgendering can decrease the chances of using condoms in sexual intercourse. A possible explanation for this is supported by the psychological mediation framework, which suggests that experiencing continuous stressors associated with the stigma against sexual and gender minorities can considerably increase levels of emotional dysregulation [58,59]. It is known from research with samples of cis people that the difficulty in regulating emotions can contribute to a reduced likelihood of using condoms in sexual intercourse [60,61]. It is suggested, therefore, that the possible emotional dysregulation that may result from experiences with misgendering may contribute to a greater likelihood of not using condoms in sexual intercourse. Although the experience of being publicly identified as transgender without the person having revealed it is not necessarily discriminatory, the continued exposure to social stigma and the constant public scrutiny of their gender expression can contribute to an increase in emotional dysregulation, leaving transgender people more susceptible to engaging in risky behaviors.

It is noteworthy that general minority stress variables from studies with cisgender lesbian, gay and bisexual (LGB) people were not significant in our study (perceived prejudice and anticipated prejudice). Stressors that are specific to transgender people (“passing” concerns and experiences with misgendering), which only affect them, were related to CPS. This leads to the conclusion that studies with transgender people from the perspective of minority stress must always take into account specific variables related to the unique experiences of transgender people. The data in this study were collected 6 years ago at a time when legislation and protective measures for gender diversity were advancing in Brazil. At present, Brazil is experiencing a major setback in these fields that affect not only the structural level but the mental health of gender and sexual minorities. Therefore, we believe that if done today, the research would show more extreme outcomes.

## 5. Conclusions

Our study complements the literature of the field and innovates, mainly because it demonstrates the impact of condom negotiation self-efficacy on condom use, and suggests macro and micro-interventions address the topic. However, some important limitations should be noted. The groups present differences between genders, mainly concerning CPS (explained by differences in sexual practices), which can affect our results. Other variables that can explain sexual behavior (resilience, coping, connection to the community, sexual frequency, and sexual abstinence) were not included in the research protocol. The cross-sectional design prevents the more accurate establishment of predictors. The sample size does not allow unrestricted generalizations about the transgender population. Non-binary people were not included in the sample, which fails to include the important diversity present in transgender communities. It is important to note that more details on the sexual partner of the participants were not included in the study. People who tend to have sex with people with penises may have a different relation to condom use than people who do not, for example [62]. Also, other safer sex/barrier practices (e.g., dental dams for oral sex, gloves for digital sex, etc.) were not discussed, which limits the range of data interpretation.

Regardless of the perceived and anticipated level of prejudice, whether higher or lower, transgender people may not engage in CPS. Factors such as CNSE, experiences of misgendering, and “passing” concerns could help to explain this, as our data demonstrated. Furthermore, other aspects not evaluated in this study could have influence on lower or higher CPS levels, such as sexual frequency and abstinence. TW sex workers have a higher sexual frequency with more sexual partnerships, which could decrease the chance of CPS (in addition to a greater chance of suffering police and street violence, which can act as confounding variables) [63,64]. Sexual abstinence (e.g., case of transgender people who do not have sex due to sexual dysfunctions or other such as surgery complications, pain, and high body shame) also could lead to lower CPS [47,65].

The set of data documented by our study can inform the formulation of public policies geared towards some of the outcomes found here. Structural interventions to reduce prejudice levels would, directly and indirectly, have a positive impact on the sexual health of transgender individuals, as some of the variables related to minority stress negatively impacted condom use. Our data suggest that TW and TM share similar risks, which justifies the need for TM to be included as policy targets. Governments need to take responsibility for monitoring and assessing prejudice through public policies in addition to actions related to the health and prevention of HIV/AIDS and other STIs.

Structural interventions to reduce levels of prejudice could directly and indirectly have a positive impact on the sexual health of transgender people, since some of the variables related to minority stress negatively impacted condom use. Governments need to take responsibility for monitoring and evaluating structural and interpersonal prejudice levels by several public policies in addition to actions related to health and prevention of HIV/AIDS and other STIs.

Ensuring greater protection and security for transgender people, who are burdened by a plethora of systematic vulnerabilities, could facilitate access to housing and labor market opportunities. These aspects may be associated with a higher probability of sexual work, which in certain contexts may contribute to lower levels of CPS in transgender people, especially TW. It would be useful to foster the creation of public shelters or specific housing aids, along with broadening incentive for private companies to offer technical qualifications and vacancies for transgender people.

Education campaigns to foster the debate on gender and sexuality in schools and universities would be elementary to promote respect for gender diversity, especially taking into account the political violence towards gender and sexual minorities in countries such as Brazil. Previous evidence indicates that receiving qualified and accurate information on gender and sexual minorities can influence the mitigation of prejudice at cognitive and behavioral levels [66,67].

In addition to changes in the macro level, clinical interventions should be developed to in order to address some of the outcomes described in our study. Our main concern is CNSE, which was strongly associated with lower levels of CPS in transgender people’s sexual relationships. There are numerous psychological resources which could be used to train assertiveness skills, such as social skills training programs [68] or dialectical behavioral therapy (DBT) skills training [69].

We also consider necessary the development of psychological interventions to mitigate the deleterious effects of rumination and “passing” concerns among transgender people. Previous literature has indicated that the most effective intervention to relieve both gender dysphoria and rumination are gender affirmation medical procedures [52]. Psychological counseling could facilitate access to these procedures by discussing of pros and cons, as well as providing guidance on how and where to accomplish these procedures.

However, Brazil and other countries face several structural limitations regarding the the access to these procedures, as most of transgender individuals cannot afford them or have to wait many years for a medical appointment in the public health system to be accomplished. Therefore, other possibilities need to be considered. Studies have suggested that mindfulness-based practices can help to reduce rumination [70,71]. Mindfulness is the ability to redirect attention and be aware of the present moment, without trying to modify or avoid it [72]. The provision of culturally adapted mindfulness protocols, both in individual and group modalities, could aid to mitigate body rumination [73]. The combination of macro and micro-level interventions has potential to improve the outcomes highlighted by our study and foster overall well-being of transgender people [74].

## Figures and Tables

**Table 1:** Sociodemographic profile.

	Statistical Distribution		
Measures	Groups	*N*	%
Gender identity	Transgender women	192	73.8
	Transgender men	68	26.2
			
State	Rio Grande do Sul	79	30.4
	São Paulo	181	69.6
			
Race/ethnicity	Black	15	5.8
	White	184	70.8
	*Pardo*	54	20.8
	Indigenous	1	0.4
	Yellow/Asian	6	2.3
Level of education	No formal education	4	1.5
	Elementary education	20	7.7
	High school education	162	62.3
	Higher education	59	22.7
	Postgraduate degree	15	5.8
Marital status	Single	182	70
	Common-law marriage	39	15
	Married	33	12
	Divorced	6	2.3

**Table 2:** Differences between TM and TW.

Measures			TM		TW					
	*M*	*SD* *(n)*	*M*	*SD* *(n)*	*M*	*SD* *(n)*	*t*	*p* *	*d*	*95% IC*
CPS (insertive oral sex)	1.46	0.92 (231)	1.30	0.84 (60)	1.52	0.94 (171)	1.72	0.088	0.57	[0.54, 0.06]
CPS (receptive oral sex)	1.78	1.15 (229)	1.52	1.10 (59)	1.87	1.15 (170)	2.00	0.047	0.31	[0.61, 0.01]
CPS (insertive penetration)	1.86	1.24 (230)	2.05	1.35 (60)	1.78	1.20 (170)	−1.33	0.186	0.22	[0.08, 0.51]
CPS (receptive penetration)	2.40	1.38 (230)	1.25	0.73 (60)	2.81	1.32 (170)	8.66	0.000	1.30	[1.62, 0.99]
CPS total score	7.51	3.43 (226)	6.15	3.15 (59)	7.99	3.41 (167)	3.63	0.000	0.65	[0.85, 0.25]
CNSE item 1	5.53	2.12 (226)	4.78	2.69 (60)	5.80	1.81 (166)	2.73	0.008	0.49	[0.79, 0.19]
CNSE item 2	5.03	2.30 (217)	4.32	2.75 (57)	5.28	2.07 (160)	2.43	0.017	0.42	[0.73, 0.12]
CNSE item 3	5.40	2.10 (222)	4.74	2.69 (58)	5.64	1.80 (164)	2.35	0.021	−0.62	[−0.74, −0.13]
CNSE item 4	5.25	2.24 (215)	4.67	2.74 (56)	5.45	2.01 (159)	1.94	0.006	0.35	[0.66, 0.04]
CNSE item 5	5.33	2.11 (218)	4.89	2.65 (57)	5.48	1.87 (161)	1.54	0.126	0.28	[0.58, 0.02]
CNSE item 6	5.56	2.05 (219)	5.00	2.61 (57)	5.76	1.78 (162)	2.03	0.045	0.38	[0.68, 0.07]
CNSE item 7	5.52	2.06 (221)	4.85	2.63 (57)	5.75	1.77 (164)	2.37	0.020	0.44	[0.75, 0.14]
CNSE total score	37.67	13.68 (212)	32.96	17.59 (55)	39.31	11.64 (157)	2.49	0.015	0.47	[0.78, 0.16]
Self-esteem	18.97	6.17 (178)	17.11	6.74 (44)	19.58	5.88 (134)	2.32	0.034	0.41	[0.74, 0.06]
Experiences with misgendering	2.79	1.30 (225)	2.26	1.10 (60)	2.99	1.32 (165)	4.10	0.000	0.58	[0.88, 0.28]
Anticipated prejudice	3.30	2.72 (217)	3.63	2.82 (58)	3.17	2.69 (159)	−1.08	0.283	0.17	[0.13, 0.47]
Perceived prejudice	4.90	2.07 (182)	4.69	2.20 (45)	4.97	2.03 (137)	0.76	0.451	0.14	[0.47, 0.20]

Note: CPS = condom-protected sex; CNSE = condom negotiation self-efficacy; CNSE item 1 = “I can ask a new partner to use a condom”; CNSE item 2 = “I can ask a partner with whom I didn’t use a condom to start doing this”; CNSE item 3 = “I can refuse sex when I don’t have condoms available”; CNSE item 4 = “I can make a partner use a condom even when I’m drunk or drugged”; CNSE item 5 = “I can make a partner use a condom even when he/she doesn’t want to”; CNSE item 6 = “I can ask a partner who knows I am transgender to use condoms”; CNSE item 7 = “I can ask any partner to use a condom”; TM = transgender men; TW = transgender women. * Group differences corrected for false discovery rate.

**Table 3:** Pearson correlation matrix (*n* = 122 to 167).

	TW	(1)	(2)	(3)	(4)	(5)	(6)	(7)	(8)	(9)	(10)	(11)	(12)	(13)	(14)	(15)	(16)	(17)
TM																		
(1) CPS total score		1	0.73 **	0.77 **	0.63 **	0.80 **	0.21 **	0.20 *	0.23 **	0.18 *	0.18 *	0.20 *	0.21 **	0.23 **	0.10	0.17 *	−0.00	−0.03
(2) CPS (insertive oral sex)		0.79 **	1	0.58 **	0.31**	0.35**	0.04	0.06	0.11	0.05	0.06	0.08	0.06	0.09	0.10	0.15	−0.7	−0.02
(3) CPS (receptive oral sex)		0.85 **	0.65 **	1	0.15 **	0.54 **	0.19 **	0.16 *	0.20 *	0.18 *	0.18 *	0.20 *	0.20 *	0.21 **	0.06	0.11	−0.06	−0.03
(4) CPS (insertive penetration)		0.81 **	0.47 **	0.53 **	1	0.37 **	−0.02	0.06	0.03	0.02	0.02	−0.03	0.02	0.03	0.06	0.20 **	0.13	−0.01
(5) CPS (receptive penetration)		0.60 **	0.37 **	0.41 **	0.30 **	1	0.35 **	0.29 **	0.31 **	0.25 **	0.27 **	0.32 **	0.30 **	0.34 **	0.04	0.03	−0.06	−0.02
(6) CNSE item 1		0.39 **	0.25	0.22	0.46 **	0.22	1	0.65 **	0.80 **	0.72 **	0.78 **	0.78 **	0.83 **	0.89 **	0.17 *	−0.18 *	−0.03	0.07
(7) CNSE item 2		0.24	0.23	0.02	0.29 *	0.20	0.81 **	1	0.64 **	0.65 **	0.72 **	0.72 **	0.76 **	0.83 **	0.15	−0.07	0.02	0.04
(8) CNSE item 3		0.37 **	0.21	0.25	0.45 **	0.20	0.81 **	0.64 **	1	0.69 **	0.70 **	0.71 **	0.77 **	0.87 **	0.17	−0.16 *	−0.01	0.04
(9) CNSE item 4		0.41 **	0.27 *	0.29 *	0.47 **	0.18	0.85 **	0.74 **	0.87 **	1	0.82 **	0.78 **	0.75 **	0.87 **	0.20 *	−0.16 *	−0.03	0.07
(10) CNSE item 5		0.40**	0.25	0.27*	0.47**	0.14	0.88**	0.79**	0.84**	0.95**	1	0.84**	0.80**	0.91**	0.11	−0.12	−0.03	0.09
(11) CNSE item 6		0.38 **	0.24	0.21	0.45 **	0.22	0.96 **	0.78 **	0.87 **	0.89 **	0.93 **	1	0.87 **	0.92 **	0.22 *	−0.15	−0.14	−0.00
(12) CNSE item 7		0.39 **	0.25	0.21	0.46 **	0.24	0.95 **	0.77 **	0.85 **	0.91 **	0.92 **	0.98 **	1	0.93 **	0.18 *	−0.10	−0.06	0.03
(13) CNSE total score		0.40 **	0.27 *	0.23	0.47 **	0.23	0.95 **	0.84 **	0.89 **	0.95 **	0.96 **	0.98 **	0.97 **	1	0.21 *	−0.16 *	−0.05	0.06
(14) Self-esteem		0.26	0.19	0.17	0.19	0.28	0.10	0.03	0.29	0.28	0.20	0.18	0.16	0.24	1	0.05	−0.36 **	-0.23 **
(15) Experiences with misgendering		0.15	0.13	0.24	−0.02	0.16	−0.04	−0.11	0.03	−0.03	−0.05	−0.03	−0.08	−0.1	0.22	1	0.20 *	0.05
(16) Anticipated prejudice		0.28 *	−0.19	−0.21	−0.28	−0.12	−0.14	−0.06	−0.24	−0.13	−0.19	−0.17	−0.16	−0.16	−0.27	−0.01	1	0.39 **
(17) Perceived prejudice		0.01	−0.02	0.01	−0.03	0.12	0.09	0.03	−0.15	−0.01	0.05	0.06	0.03	0.01	−0.22	0.09	0.44 **	1

Note: CPS = condom-protected sex; CNSE = condom negotiation self-efficacy; CNSE item 1 = “I can ask a new partner to use a condom”; CNSE item 2 = “I can ask a partner with whom I didn’t use a condom to start doing this”; CNSE item 3 = “I can refuse sex when I don’t have condoms available”; CNSE item 4 = “I can make a partner use a condom even when I’m drunk or drugged”; CNSE item 5 = “I can make a partner use a condom even when he/she doesn’t want to”; CNSE item 6 = “I can ask a partner who knows I am transgender to use condoms”; CNSE item 7 = “I can ask any partner to use a condom”. * *p* <0.05; ** *p <* 0.01.

**Table 4:** Differences in CPS in TM and TW with and without “passing” concerns.

Measures	“Passing” Concerns							
	No		Yes					
	*M*	*SD (n)*	*M*	*SD (n)*	*T*	p *	*d*	*95% IC*
CPS (insertive oral sex)	1.29	0.74 (89)	1.62	0.97 (53)	2.14	0.02	−0.40	[0.05, −0.74]
CPS (receptive oral sex)	1.62	1.07 (87)	1.86	1.18 (51)	1.23	0.22	−0.22	[0.13, −0.56]
CPS (insertive penetration)	1.56	1.11 (87)	2.20	1.32 (51)	3.01	0.03	−0.54	[−0.18, −0.09]
CPS (receptive penetration)	2.07	1.36 (88)	2.67	1.38 (53)	2.51	0.01	−0.44	[−0.10, −0.78]
CPS total score	6.53	2.94 (87)	8.50	3.71 (50)	3.42	0.01	−0.63	[−0.28, −0.99]

Note: CPS = condom-protected sex; TM = transgender men; TW = transgender women. * Group differences corrected for false discovery rate.

**Table 5:** Multiple linear regression for CPS in TM and TW.

CPS (*n* = 97)					
Variables	*B*	*95% IC*	*Β*	*T*	*p*
Constant	−0.54	[−3.35, 2.27]	-	0.38	0.702
CNSE	0.10	[0.06, 0.13]	0.46	5.19	0.000
Self-esteem	0.01	[−0.10, 0.10]	0.02	0.02	0.984
Experiences with misgendering	0.49	[0.03, 0.94]	0.19	2.11	0.038
“Passing” concerns	2.16	[0.088, 3.44]	0.31	3.34	0.001
R^2^_adj_ = 0.29					

Note: CPS = condom-protected sex; CNSE = condom negotiation self-efficacy; TM = transgender men; TW = transgender women.

## Data Availability

The data is not publicly available because it is partially derived from confidential medical records. If anyone would like to have access to it for research purposes (e.g., systematic reviews or meta-analyses), please contact the corresponding author via e-mail.
